# Causes of Infertility: Diagnosis and Management With Microfluidic Sperm Sorter

**DOI:** 10.7759/cureus.27674

**Published:** 2022-08-04

**Authors:** Saurabh Chauhan, Akash More, Deepti Shrivastava, Ujwal Gajbe

**Affiliations:** 1 Clinical Embryology, School of Allied Health Sciences, Datta Meghe Institute of Medical Sciences, Wardha, IND; 2 Senior Embryologist, Datta Meghe Institute of Medical Sciences, Wardha, IND; 3 Obstetrics and Gynaecology, Jawaharlal Nehru Medical College, Datta Meghe Institute of Medical Sciences, Wardha, IND; 4 Anatomy, Datta Meghe Medical College, Datta Meghe Institute of Medical Sciences, Nagpur, IND

**Keywords:** oligoasthenozoospermia, frozen oocyte, microfluidic, spillage, infertility

## Abstract

This case study refers to a three-and-a-half-year married couple who visited an infertility clinic and are facing infertility for two-and-a-half years. They started in vitro fertilization/assisted reproduction technology (IVF/ART) procedures from December 2021. In 2019, the couple underwent infertility therapy in Wardha, India, including three intra‐uterine insemination (IUI) treatments which failed. The couple was then recommended the intracytoplasmic sperm injection (ICSI) procedure in which the semen sample was separated using a microfluidic sperm sorter for separating good quality sperms. There was no past medical history of both of the partners. Since she was infertile, the desire to have a child was extremely strong. Sadness, helplessness, bewilderment, and frustration are all common feelings associated with infertility. The left fallopian tube delays spillage in females and oligoasthenozoospermia in males; women were detected with secondary infertility and male sperm samples were sorted using a microfluidic sperm sorter and fertilized frozen oocytes for ICSI. As the male partner, in this case, had oligoasthenozoospermia, he was prescribed multivitamins and other medications. After ICSI, the blastocyst formation was good with the use of a microfluidic sperm sorter.

## Introduction

Assisted reproduction technology (ART) is highly effective for infertility patients. Infertility is a major clinical problem that acts on 8% to 12% of couples all over the world [1]. According to the World Health Organization (WHO), infertility has been known as a significant health issue that might lead to stress [1]. Lack of understanding about reproductive capacity is a major cause of fertility delay and it increases the risk of infertility. However, accurate knowledge of reproductive disorders is essential for better fertility outcomes. Currently, there is a lack of knowledge concerning infertility worldwide [2].

Awareness of infertility, including male and female risk factors, is an essential step toward maintaining fertility through lifestyle changes. Fertility knowledge is related to education rather than personal fertility or parenthood experience. As a result, educational interventions are recommended as a starting point for health promotion programs. Promoting fertility health is not a common trend in the literature [3]. People are typically unaware of the biological components of conception; they frequently overestimate the possibilities of pregnancy during the time of ovulation and have a poor understanding of when women are most fertile, according to data [4].

Infertile couples frequently encounter marital discord, especially when they are under pressure to make medical decisions. Clinical depression is more common among women trying to conceive, with rates comparable to those with heart disease or cancer. Emotional stress and marital troubles are more common in couples where the male partner is infertile [5-8].

The primary goal of blastocyst culture was to improve in vitro fertilization (IVF) success ratios through better embryo selection. Blastocyst culture has also been used to select the most viable embryos in a cohort, decreasing the number of embryos transplanted and, as a result, decreasing the number of multiple gestations [9].

Microfluidic sperm sorting is a novel method that uses a microfluidic device with four chambers (A, B, C, and D); sperms are put in one chamber and a buffer solution in another, with the sperms traveling through microchannels [10]. The microfluidic device is made of polydimethylsiloxane (PDMS), Pyrex, silicone, quartz, and borosilicate glass. The polymer PDMS is a popular choice for microchannel fabrication. It isolates progressive motile sperms from a semen sample which runs through microchannels of microfluidic devices by applying hydrostatic pressure. The exceptionally motile sperms are gathered in one compartment and then used [11]. Another microfluidic device arrangement for determining the total amount of sperm and the percentage of motile sperm is based on the random swimming and sedimentation principles of sperm. This platform can also tell you how many motile and immotile sperm there are [12]. Improved laboratory and clinical outcomes have been reported with microfluidic sperm selection for ICSI, such as the possibility of producing more high-quality blastocysts, euploid blastocysts, and surplus high-quality blastocysts to be frozen for future use [13]. This study evaluates a couple facing infertility for two-and-a-half years with ongoing treatment with ICSI with good quality blastocyst formation using a microfluidic sperm sorter.

## Case presentation

This case report refers to a three-and-a-half-year married couple - a 33-year-old female and a 38-year-old male who has been facing infertility for two-and-a-half years. Since December 2019, both partners had taken the treatment for infertility in Wardha with three failed cycles of intra‐uterine insemination (IUI) treatment. The couple was anxious to conceive and tried for two-and-a-half years with secondary infertility.

Medical history

Chromotubation procedure was done where in the right tube, free spillage was observed, but in the left tube, spillage was delayed. There was a spontaneous abortion after one-and-a-half weeks of marriage. Semen analysis of the husband was low sperm count and sperm motility, indicating oligoasthenozoospermia. It could be due to his consumption of alcohol and smoking. Both partners had no history of hypertension, tuberculosis, asthma, or cardiac disorders. The family history was negative, and the couple had no previous mental or psychiatric disease.

Clinical findings

A hysteroscopy procedure was done for the female patient; the uterine cavity was normal, bilateral ostia were seen to appear normal, and the cervix and endocervix were also normal. A laparoscopy procedure revealed a normal uterus with bilateral normal ovaries, a normal right fallopian tube, and a left fallopian tube that showed terminal hydrosalpinx. The general condition was fair, and the temperature was afebrile.

Timeline

Both the partners started their IVF/ART for their secondary infertility in December 2019. The female patient had a history of three failed IUIs. For a general, major, and essential physical examination, the couple exhibited normal clinical findings. After this, the couple underwent IVF/ICSI procedure where the male semen sample was processed using the microfluidic sperm sorter technique. On day 5, blastocyst was formed and was transferred.

Diagnostic assessment

A hysterosalpingogram test (HSG) was done on the patient. The uterine cavity was normal, Bilateral ovaries were normal, normal spillage was seen in the right fallopian tube, and delayed spillage was seen in the left fallopian tube. The complete blood count (CBC) test was normal. The anti-Müllerian hormone (AMH) value of the patient was 3.5 ng/ml. The couple was diagnosed with secondary infertility, tubal factor in the female partner (delayed spillage) with oligoasthenozoospermia in the male partner.

Therapeutic intervention

Both the partners were advised to pursue IVF/ICSI treatment. For ICSI, the male semen sample was separated using a microfluidic sperm sorter and was fertilized with a frozen oocyte. Before collecting the semen sample, administrative and therapeutic interventions were included such as medications like coenzyme Q10, vitamin C, vitamin B6, niacinamide, l-carnitine, l-tartrate, vitamin E, and zinc which increase the sperm count and motility.

Followup and outcomes

Clinical and patient assessed outcomes: The case is of a couple with blastocyst formation through ICSI/IVF procedure with 3 IUI failures earlier. After using microfluidic sperm sorting for ICSI, the blastocyst formation was considered good.

## Discussion

Parenthood is seen as a real measure of a good marital life in India. In India, like in other underdeveloped nations, infertility is a taboo subject, and childlessness carries a lot of societal stigmas [14]. Every time females are criticized for infertility, new research shows that males are equally responsible for the problem which leads to depression, trauma, and emotional stress which can affect the individual [15,16]. This study reveals that the female had delayed spillage which refers to fallopian tube abnormality [17,18]. The channel for sperm to reach the eggs, as well as the road back to the uterus for the fertilized egg, is obstructed if a fallopian tube is closed [17].

As per our study, the failure of IUI (three times) may be due to the blockage of the fallopian tube. HSG is inferior to laparoscopy and dye testing [17]. This study revealed that the male patient had oligoasthenozoospermia [19]. Stress has also been shown to have a deleterious impact on testosterone, progesterone, cortisol, and prolactin levels [18]. Men's sperm motility and semen volume have been found to decline as they grow older [20]. Sperm is made up of unsaturated fatty acids, which are vulnerable to free radical damage caused by lipid peroxidation, which can be caused by physiological and environmental causes [21]. The reason for low sperm count can be the consumption of alcohol and smoking [22]. It also exhibits relatively few motile sperm and aberrant morphology in our investigation [23]. After ICSI transfer, a high proportion of blastocyst formation was discovered, with about 70% developed in vitro and then fertilized by ICSI, having the ability to evolve to the blastocyst stage [24]. When compared to IUI, blastocyst development in ICSI was relatively high in this study [25]. Primary infertility is more prevalent than secondary infertility [26], insufficient care during earlier pregnancies or past abortions that resulted in pelvic infections could be the reason for the rise in secondary infertility patients [17,27].

Before fertilization, selecting high-quality oocytes is just as crucial as selecting high-quality sperms. The quality of an oocyte can influence the outcome of fertilization and the health of the resulting embryo [28]. Microfluidic technologies, particularly in ART, are still a relatively new subject. Few successful sperm counting and sorting methods have been described in the literature. However, constructing and commercializing microfluidic devices, as well as gaining acceptance from relevant employees and users, face some challenges [12]. Sperm with improved motility and dramatically reduced DNA fragmentation are produced using a microfluidic system for sperm sorting. With little effort on the part of the technician, the device produces a highly motile population of sperm [13].

## Conclusions

Our case is of secondary infertility in which the female patient’s treatment is ongoing and there may be a chance of blockage of the fallopian tube. The male partner had oligoasthenozoospermia due to consumption of alcohol and smoking. The patient underwent a microfluidic sperm sorting process which helped in the selection of good quality sperms used for ICSI and the outcome of the blastocyst was very good.
